# Supplementary material for: Engineering Af1521 improves ADP-ribose binding and identification of ADP-ribosylated proteins
Source: Nat Commun. 2020 Oct 15;11:5199. doi: 10.1038/s41467-020-18981-w (PMC7566600; doi:10.1038/s41467-020-18981-w)
Supplement: Supplementary file 1 — Supplementary Information [file 41467_2020_18981_MOESM1_ESM.pdf]

Supplementary Information

Engineering Af1521 improves ADP-ribose binding and identification of ADP-ribosylated proteins

Kathrin Nowak[1, 2], Florian Rosenthal[1], Tobias Karlberg[3], Mareike Bütepage[4], Ann-Gerd Thorsell[3], Birgit Dreier[5], Jonas Grossmann[6, 7], Jens Sobek[6], Ralph Imhof[1], Bernhard Lüscher[4], Herwig Schüler[3], Andreas Plückthun[5], Deena M. Leslie Pedrioli[1] and Michael O. Hottiger[1]

[1]Department of Molecular Mechanisms of Disease, University of Zurich, Switzerland
[2]Molecular Life Science PhD Program of the Life Science Zurich Graduate School, University of Zurich, Switzerland
[3]Department of Biosciences and Nutrition, Karolinska Institute, Huddinge, Sweden
[4]Institute of Biochemistry and Molecular Biology, RWTH Aachen University, Germany
[5]Department of Biochemistry, University of Zurich, Switzerland
[6]Functional Genomics Center Zurich, ETH Zurich and University of Zurich, Switzerland
[7]SIB Swiss Institute of Bioinformatics, Quartier Sorge - Batiment Amphipole, Lausanne, Switzerland

Supplementary Figures 1 – 5

Supplementary Figure 1

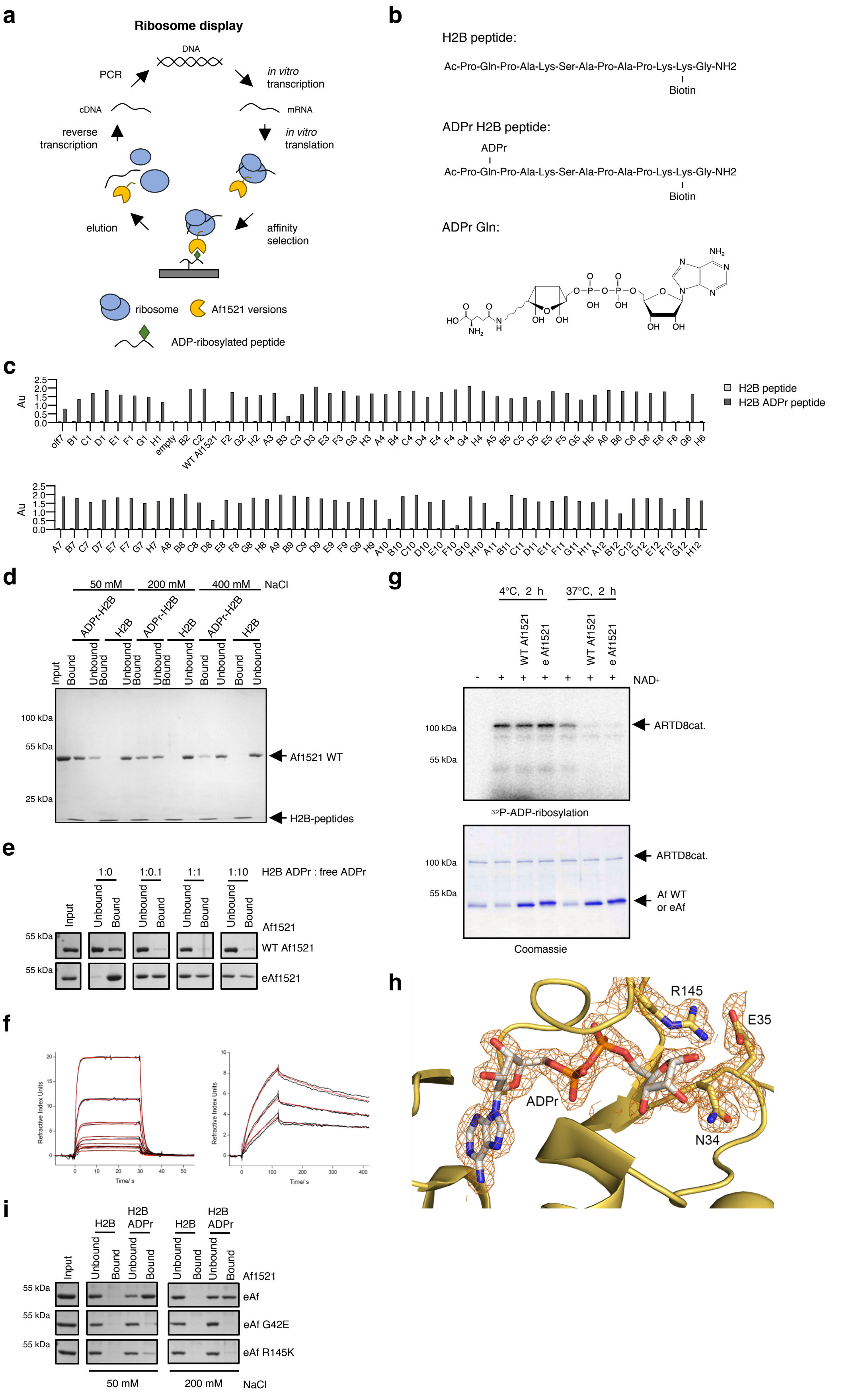

Supplementary Figure 1: Ribosome display evolves macro domain eAf1521

a Schematic illustration of the ribosome display selection cycle to evolve Af1521 macro domains with higher affinity towards ADP-ribosylation by selection with ADPr-modified H2B peptide (47). **b** Representation of the unmodified and modified H2B peptide that was synthetically ADP-ribosylated with a N-glycosidic linkage on Gln (Q) at position 2 of the peptide (47). **c** ELISA analysis of eAf1521 variants against the unmodified and ADPr H2B peptide. The ELISA was performed after the 4th round of error-prone PCR followed by selection using ribosome display. AU, absorbance unit. **d** Exemplary SDS-PAGE gel of pull-down experiment using unmodified and ADP-ribosylated H2B peptides (biotinylated and bound to streptavidin Sepharose beads) of Af1521 WT. The proteins were analyzed by SDS-PAGE followed by Coomassie staining. The whole SDS-PAGE gel confirms equal concentration of peptides are used and that the elution was sufficient for the binding assay. The experiment was repeated independently ($n=3$) with similar results. **e** Pull-down experiment using ADP-ribosylated H2B peptides (biotinylated and bound to streptavidin Sepharose beads) of WT Af1521 and eAf1521 under increasing ADPr concentration. The proteins were analyzed by SDS-PAGE followed by Coomassie staining. The experiment was repeated independently ($n=2$) with similar results. **f** Panel left: SPR sensorgrams for binding of ADPr (2500 – 39 nM) on immobilized WT Af1521. Panel right: Sensorgrams for binding of ADPr (25 - 6.25 nM) on immobilized eAf1521. **g** Demodification assay of radiolabeled automodified ARTD8cat. ADP-ribosylated ARTD8cat was detected by autoradiography. The experiment was repeated independently ($n=2$) with similar results. **h** View of the eAf1521 bound ADP-ribose around the ADP-ribose as well as side chains N34, E35 and R145 (omit map contoured at 1.5 σ). **i** Pull-down experiment using unmodified and ADP-ribosylated H2B peptides (biotinylated and bound to streptavidin Sepharose beads) of eAf1521, eAf1521-G42E and eAf1521-R145K. Input lanes are the same for all measured conditions. The proteins were analyzed by SDS-PAGE followed by Coomassie staining. The experiment was repeated independently ($n=2$) with similar results. Source data are provided as a Source Data file.

Supplementary Figure 2

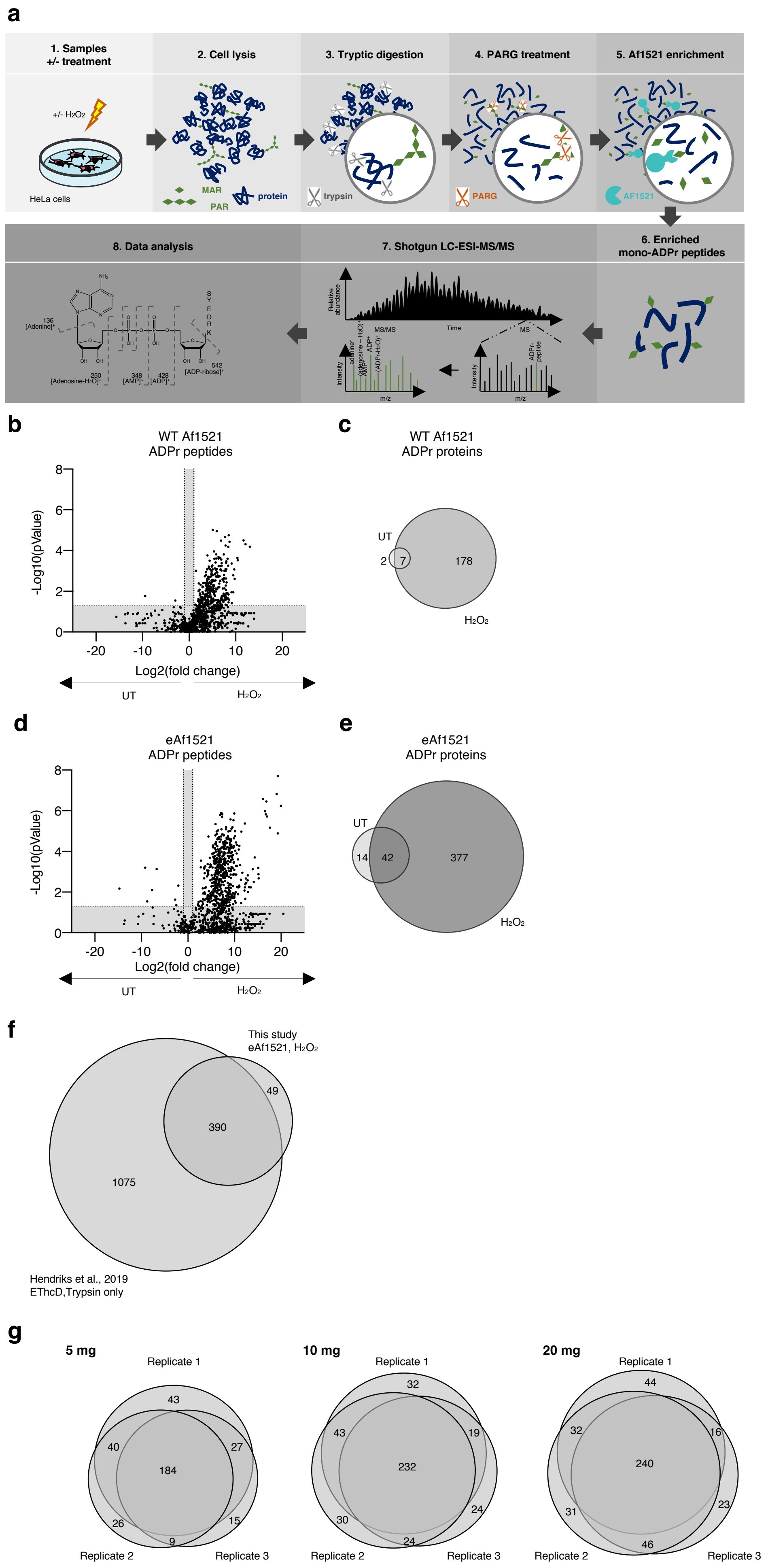

Supplementary Figure 2: Comparison of MS-based identification of ADP-ribosylated peptides and proteins using WT Af1521 or eAf1521

a Schematic drawing of the used MS workflow adapted form (26). **b** Volcano plot comparing ADP-ribosylated peptides in either untreated or H_2O_2-treated HeLa cells lysate enriched with WT Af1521. The statistical analysis and *p*-value calculation was performed with two-tailed Student's *t*-test, the black lines represent foldchange > 2 and *p*-value < 0.05. **c** Venn diagram depicting the overlap of ADP-ribosylated protein groups in either untreated or H_2O_2-treated HeLa cells enriched with WT Af1521. **d** Volcano plot comparing ADP-ribosylated peptides in either untreated or H_2O_2-treated HeLa cells lysate enriched with eAf1521. **e** Venn diagram displaying the overlap of ADP-ribosylated protein groups in either untreated or H_2O_2-treated HeLa cells enriched with eAf1521. The statistical analysis and *p*-value calculation was performed with two-tailed Student's *t*-test, the black lines represent foldchange > 2 and *p*-value < 0.05. **f** Venn diagram depicting the overlap of ADP-ribosylated proteins in H_2O_2-treated HeLa cells lysate in different ADPr proteomic studies (27). For comparison with Hendriks et al. 2019 dataset only the closest experimental conditions Trypsin and EThcD were included. **g** Venn diagrams representing the biological replicates of ADP-ribosylated protein groups enriched with eAf1521 using different starting material (5 mg, 10 mg and 20 mg). Source data are provided as a Source Data file.

Supplementary Figure 3

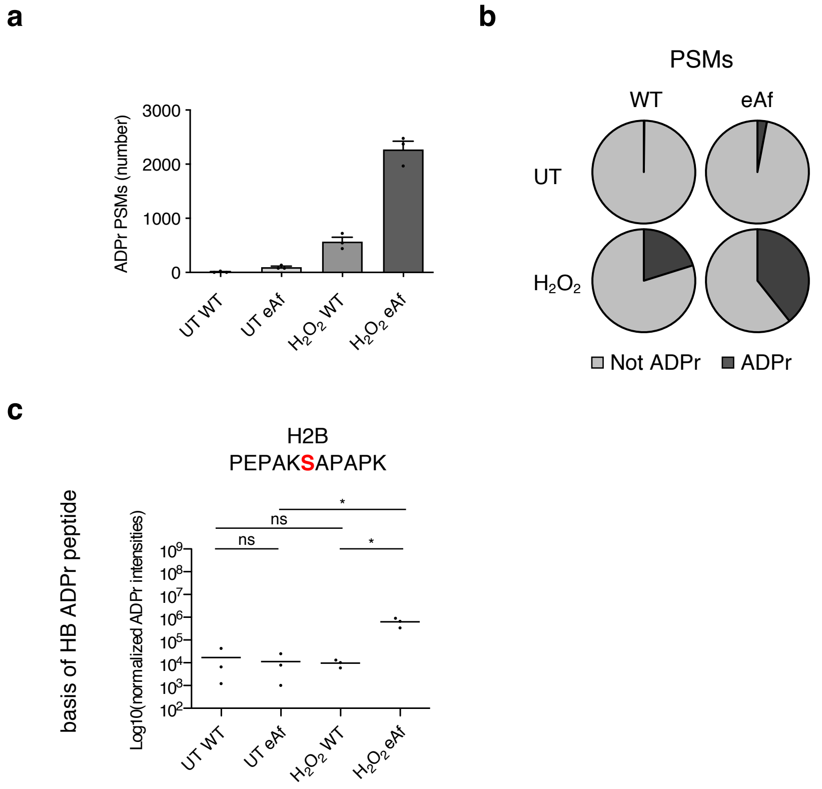

Supplementary Figure 3: Intensity of PSM is enhanced when ADP-ribosylated peptides are enriched with eAf1521

a Bar plot representing the number of identified ADPr PSMs in untreated and H_2O_2-treated HeLa cell lysates enriched with either WT Af1521 or eAf1521. Data are presented as mean with SEM (*n*=3 biochemical independent samples). **b** Pie charts of all identified and ADPr PSMs in untreated and H_2O_2-treated HeLa cell lysates enriched with either WT Af1521 or eAf1521. **c** Scatter plot depicting MS1 signal intensities of ADPr H2B peptide in untreated and H_2O_2-treated HeLa cell lysates enriched with either WT Af1521 or eAf1521 (*n*=3 biochemical independent samples). The statistical analysis and *p*-value calculation was performed with two-tailed Student's *t*-test (*$p<0.05$, ns = not significant). Source data are provided as a Source Data file.

Supplementary Figure 4

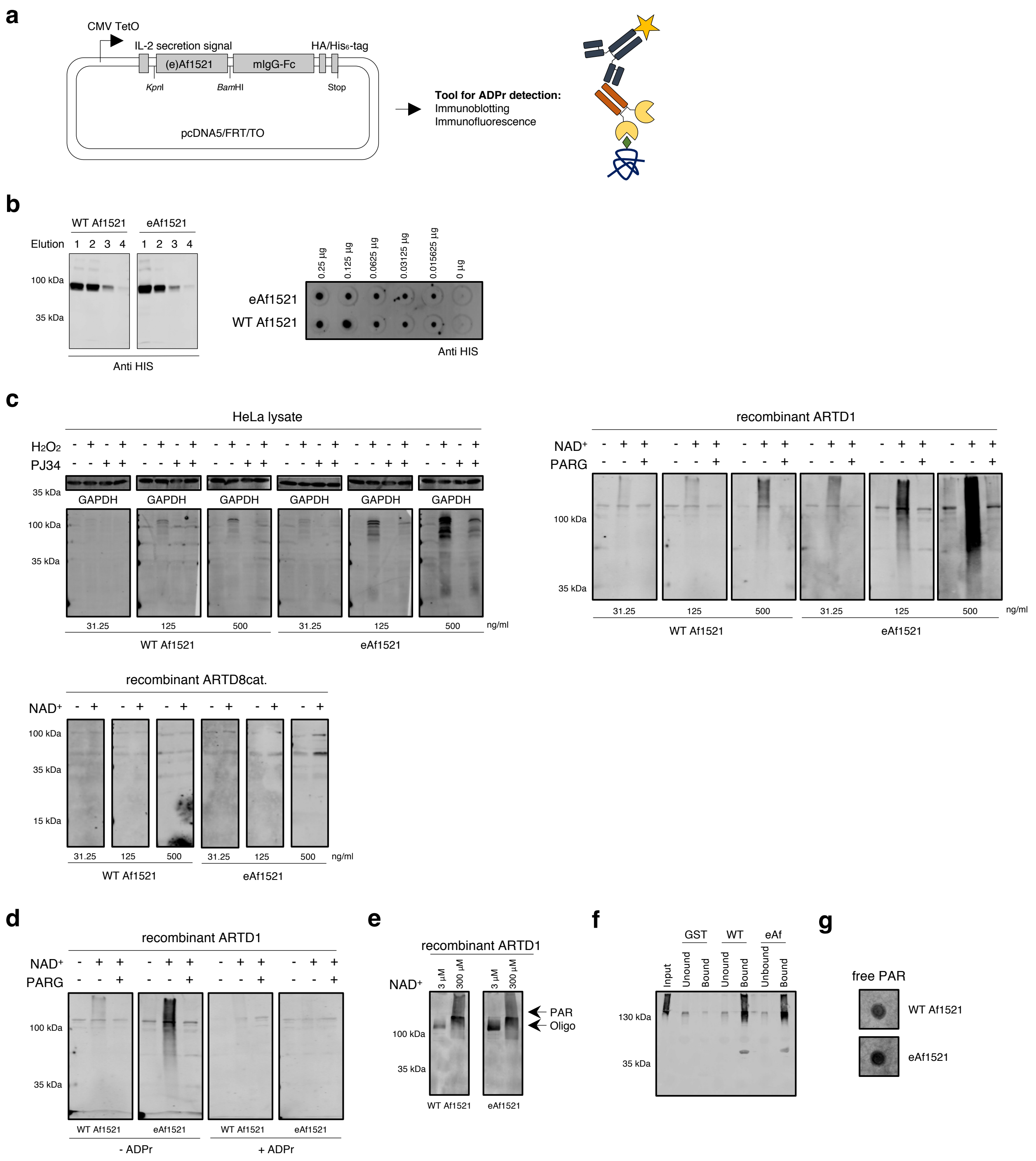

Supplementary Figure 4: Fc-WT Af1521 and eAf1521 as a tool for Western Blot analysis

a Schematic illustration of the generation of an ADP-ribosylation detection tool by fusing an Fc-fragment to WT Af1521 or eAf1521. **b** Dot blot analysis of purified Fc-WT Af1521 and Fc-eAf1521. The Fc fusion domains were detected using the His-tag. The experiment was repeated independently ($n=2$) with similar results. **c** Immunoblot analyses of untreated and H_2O_2-treated HeLa cells, including PJ34 treatment as control for ADP-ribosylation inhibition; unmodified, poly-ADP-ribosylated and mono-ADP-ribosylated ARTD1 and unmodified and mono-ADP-ribosylated ARTD8 with different concentrations of Fc-eAf1521 and Fc-WT Af1521. Detection was performed with IRDye 680 goat anti-mouse antibody. Immunoblot analyses to be compared were performed at the same time and with the same exposure. The experiment was repeated independently ($n=2$) with similar results. **d** Competition of Fc-WT Af1521 or Fc-eAf1521 with 1 μM ADPr in immunoblot analysis of unmodified, poly-ADP-ribosylated and mono-ADP-ribosylated ARTD1. Detection was performed with IRDye 680 goat anti-mouse antibody. Immunoblot analyses to be compared were performed at the same time and with the same exposure. The experiment was repeated independently ($n=2$) with similar results. **e** Dot blot analyses of free PAR chains with either Fc-eAf1521 or Fc-WT Af1521. Immunoblot analyses to be compared were performed at the same time and with the same exposure. Detection was performed with IRDye 680 goat anti-mouse antibody. The experiment was repeated independently ($n=3$) with similar results. **f** Immunoblot analyses of oligo-ADP-ribosylated and poly-ADP-ribosylated ARTD1 with either Fc-eAf1521 or Fc-WT Af1521. The automodification reaction of ARTD1 was performed with either 3 μM or 300 μM NAD^+ for 30 min at 37°C. Immunoblot analyses to be compared were performed at the same time and with the same exposure. Detection was performed with IRDye 680 goat anti-mouse antibody. The experiment was repeated independently ($n=2$) with similar results. **g** Pull-down experiment using GST-Af1521 WT, GST-eAf1521 and GST of poly-ADP-ribosylated ARTD1. Only 10% of input and unbound fractions were loaded. The proteins were detected by immunoblot analysis stained with 10H antibody (PAR antibody) and followed by IRDye 680 goat anti-mouse antibody. The experiment was repeated independently ($n=2$) with similar results. Source data are provided as a Source Data file.

Supplementary Figure 5

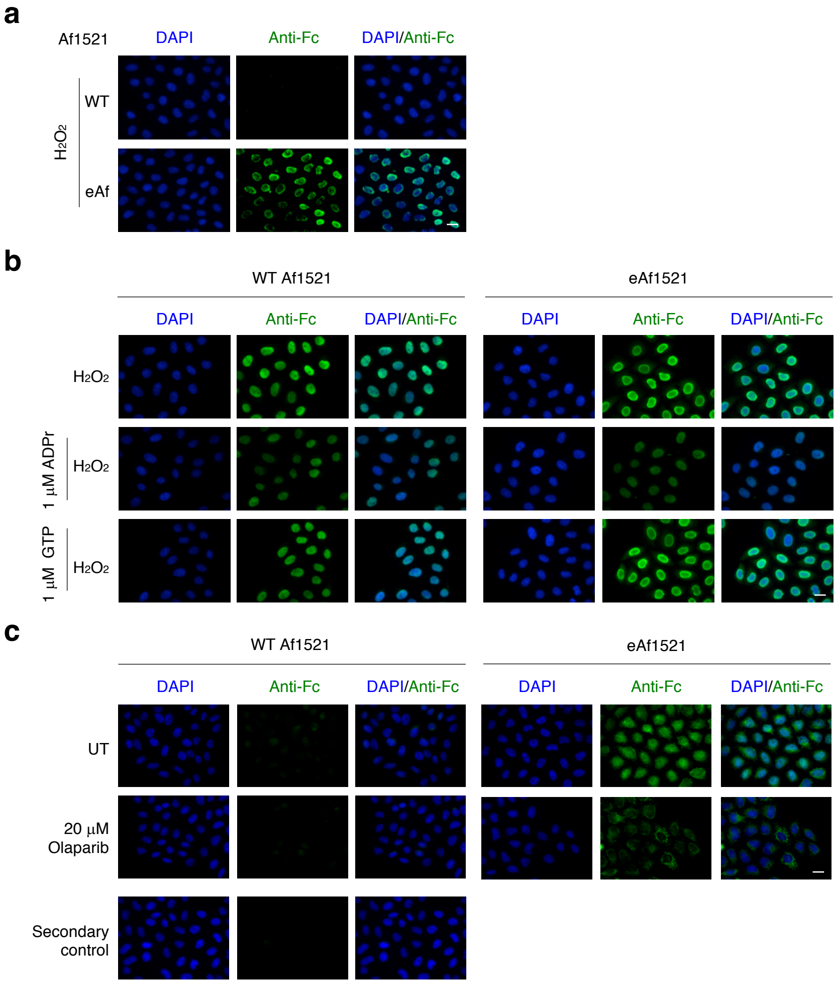

Supplementary Figure 5: Fc-WT Af1521 and Fc-eAf1521 as a tool for immunofluorescence analysis
a Immunofluorescence analysis of H_2O_2-treated HeLa cells with Fc-eAf1521 and Fc-WT Af1521. Detection was performed with an Alexa 488-labeled goat anti-mouse antibody. Signal intensity of Fc-WT Af1521 was set as background. Scale bar, 20 μm. **b** Immunofluorescence analysis of H_2O_2-treated HeLa cells with Fc-eAf1521 and Fc-WT Af1521, followed by Alexa 488-labeled goat anti-mouse antibody. Competition of Fc-WT Af1521 or Fc-eAf1521 with either 1 μM ADPr or 1 μM GTP. Scale bar, 20 μm. **c** Immunofluorescence analysis of untreated or Olaparib-treated HeLa cells with Fc-eAf1521 and Fc-WT Af1521, followed by Alexa 488-labeled goat anti-mouse antibody. Scale bar, 20 μm.
