# Supplementary material for: Engineering Af1521 improves ADP-ribose binding and identification of ADP-ribosylated proteins
Source: Nat Commun. 2020 Oct 15;11:5199. doi: 10.1038/s41467-020-18981-w (PMC7566600; doi:10.1038/s41467-020-18981-w)
Supplement: Supplementary file 3 — Description of Additional Supplementary Files [file 41467_2020_18981_MOESM3_ESM.docx]

Description of Additional Supplementary Files

File Name: Supplementary Data 1.

Description: MS1 intensities from label-free quantification ADPribosylome analysis of H2O2- and untreated Hela cell lysate enriched either with WT Af1521 or eAf1521. Related to Figure 2, 3 and Supplementary Figure 2, 3 For statistics a two-sited t-test was performed.

File Name: Supplementary Data 2.

Description: PSMs from ADP-ribosylome analysis of H2O2- and untreated Hela cell lysate enriched either with WT Af1521 or eAf1521. Related to Figure 2 and Supplementary Figure 2, 3

File Name: Supplementary Data 3.

Description: ADPr sites from ADP-ribosylome analysis of H2O2- and untreated Hela cell lysate enriched either with WT Af1521 or eAf1521. Related to Figure 2

File Name: Supplementary Data 4.

Description: PSMs from ADP-ribosylome analysis of H2O2-treated Hela cell lysate enriched with eAf1521 using different starting material amounts. Related to Figure 2 and Supplementary Figure 2
