# Supplementary material for: Engineering Af1521 improves ADP-ribose binding and identification of ADP-ribosylated proteins
Source: Nat Commun. 2020 Oct 15;11:5199. doi: 10.1038/s41467-020-18981-w (PMC7566600; doi:10.1038/s41467-020-18981-w)
Supplement: Supplementary file 8 — Reporting Summary [file 41467_2020_18981_MOESM8_ESM.pdf]

nature research

Corresponding author(s): Micheal O. Hottiger

Last updated by author(s): Aug 14, 2020

Reporting Summary

Nature Research wishes to improve the reproducibility of the work that we publish. This form provides structure for consistency and transparency in reporting. For further information on Nature Research policies, see Authors & Referees and the Editorial Policy Checklist .

Statistics

For all statistical analyses, confirm that the following items are present in the figure legend, table legend, main text, or Methods section.

n/a	Confirmed	
☐	☒	The exact sample size (*n*) for each experimental group/condition, given as a discrete number and unit of measurement
☐	☒	A statement on whether measurements were taken from distinct samples or whether the same sample was measured repeatedly
☐	☒	The statistical test(s) used AND whether they are one- or two-sided<br>*Only common tests should be described solely by name; describe more complex techniques in the Methods section.*
☒	☐	A description of all covariates tested
☒	☐	A description of any assumptions or corrections, such as tests of normality and adjustment for multiple comparisons
☐	☒	A full description of the statistical parameters including central tendency (e.g. means) or other basic estimates (e.g. regression coefficient) AND variation (e.g. standard deviation) or associated estimates of uncertainty (e.g. confidence intervals)
☐	☒	For null hypothesis testing, the test statistic (e.g. *F*, *t*, *r*) with confidence intervals, effect sizes, degrees of freedom and *P* value noted<br>*Give P values as exact values whenever suitable.*
☒	☐	For Bayesian analysis, information on the choice of priors and Markov chain Monte Carlo settings
☒	☐	For hierarchical and complex designs, identification of the appropriate level for tests and full reporting of outcomes
☒	☐	Estimates of effect sizes (e.g. Cohen's *d*, Pearson's *r*), indicating how they were calculated

Our web collection on statistics for biologists contains articles on many of the points above.

Software and code

Policy information about availability of computer code

Data collection	Mass spectra were acquired using a Thermo Fisher Scientific mass spectrometer operated with the Thermo Scientific Xcalibur software.<br>Immunofluoresence images were acquired using a Leica DMI6000 operated with the Leica LasX software.
Data analysis	Acquired mass spectra containing raw files were converted to Mascot generic format (MGF) using Proteome Discoverer (v2.1, Thermo Fisher Scientific).<br>The Mascot search engine (Matrix Science, version 2.5.1.3) was used for peptide and protein sequence identification.<br>Progenesis QI for Proteomics (v. 3.0.6039.34628, Nonlinear Dynamics) was used for MS1-based label-free quantification analysis.<br>For further analysis of post-translational modification localization site probability th softwares Scaffold 4 (version 4.8.4) and ScaffoldPTM (Version 3.2.0) was used invoking the site localization algorithm Ascore.<br>Statistical analysis, scatter plots, volcano plot analysis were performed using Prism 8.<br>Venn diagrams were performed with BioVenn (version 2007 - 2020).<br>For all images, brightness and contrast were adjusted using FIJI (Version 2.0.0.-rc-66/1.52b). For all images within one experiment, the same acquisition and image processing settings were used.<br>For structural data processing the softwares Buster (version 2.10.3), XDS (version November 11, 2013), AutoPROC (version June 1, 2017), Molprobity (version 4.02) and Phaser (version 2.6.0) were used.<br>Surface Plasmon Resonance measurements were evaluated using Biacore (version 2.0.3).

For manuscripts utilizing custom algorithms or software that are central to the research but not yet described in published literature, software must be made available to editors/reviewers. We strongly encourage code deposition in a community repository (e.g. GitHub). See the Nature Research guidelines for submitting code & software for further information.

Data

Policy information about availability of data

All manuscripts must include a data availability statement. This statement should provide the following information, where applicable:

- Accession codes, unique identifiers, or web links for publicly available datasets
- A list of figures that have associated raw data
- A description of any restrictions on data availability

Coordinates and structure factors have been deposited to the Protein Data Bank (PDB ID: 6FX7, https://www.rcsb.org/structure/6fx7) (Figure 1, C-D). Experimental data, statistics and model coordinates are available at rcsb.org under accession number 6fx7.
Coordinates and structure factors have been obtained form the public database Protein Data Bank (PDB ID: 2BFQ, https://www.rcsb.org/structure/2BFQ) (Figure 1, C-D).
The mass spectrometry proteomics data have been deposited to the ProteomeXchange Consortium via the PRIDE partner repository with the dataset identifier PXD016686. Figures with associated raw data: Figure 2, A-F; Suppl. Figure 2, B-G; Figure 3, A-C; Suppl. Figure 3, A-C

Field-specific reporting

Please select the one below that is the best fit for your research. If you are not sure, read the appropriate sections before making your selection.

☒ Life sciences ☐ Behavioural & social sciences ☐ Ecological, evolutionary & environmental sciences

For a reference copy of the document with all sections, see nature.com/documents/nr-reporting-summary-flat.pdf

Life sciences study design

All studies must disclose on these points even when the disclosure is negative.

Sample size	Sample size was not predetermined by statistical methods. No sample site calculation was performed. Experiments were repeated and the findings were reproduced. Mass spectrometry experiments were performed using sample sizes based on standard protocols. For robust label-free quantification analysis and statistical testing a sample size with at least n=3 was used.
Data exclusions	Peptide identifications mapping to decoy or contaminant proteins were excluded from further analysis. Identified peptide spectra matches (PSMs) were filtered on MS/MS identification score to ensure a false discovery rate of <1%, following pre-established data analysis protocol. Outliers based on clustering were removed to retain minimally n=3 biochemical replicates per condition (MS experiment with different starting materials).
Replication	All experiments (except of mass spectrometry) analysis were repeated and the findings were reproducible (n=2 or 3). All attempts at replication were successful. Mass spectrometry analyses were performed with at least n = 3 biochemical replicates per condition.
Randomization	Samples for mass spectometry analysis were randomized. Randomization for all other experiments was not applied.
Blinding	Investigators were not blinded. The results of the experiments are not prone to a potential observer bias.

Reporting for specific materials, systems and methods

We require information from authors about some types of materials, experimental systems and methods used in many studies. Here, indicate whether each material, system or method listed is relevant to your study. If you are not sure if a list item applies to your research, read the appropriate section before selecting a response.

Materials & experimental systems

n/a	Involved in the study	
☐	☒	Antibodies
☐	☒	Eukaryotic cell lines
☒	☐	Palaeontology
☒	☐	Animals and other organisms
☒	☐	Human research participants
☒	☐	Clinical data

Methods

n/a	Involved in the study	
☒	☐	ChIP-seq
☒	☐	Flow cytometry
☒	☐	MRI-based neuroimaging

Antibodies

Antibodies used	Fc-WT Af1521 and Fc-eAf1521 - Both primary binding reagents were generated in this study.<br>10H antibody (PAR antibody) produced in-house.<br>RGS(H)4 antibody (Qiagen, Cat No. 34650)

	TetraHis antibody (Qiagen, Cat No. 34670, Lot No. 160043206).<br>GAPDH antibody (1:1000, Santa Cruz Biotechnology, Cat No. sc-25778, Lot No. B0106).
Validation	The validation of the primary binding molecules Fc-WT Af1521 and Fc-eAf1521 are provided in this manuscript.<br>- Western Blot analysis, detection of recombinant ADP-ribosylated proteins, detection of ADP-ribosylation in cell lysates (controlled by inhibition and competition)<br>- Immunofluoresence analysis, detection of ADP-ribosylation in cells (controlled by inhibition and competition)<br>The 10H antibody was validated before and is used in several publications (Bisceglie et al. 2017, Bartolomei et al. 2016, Andersson et al. 2016).<br>Sensitivity validated by supplier for TetraHis, RGS(H)4 and GAPDH antibody.

Eukaryotic cell lines

Policy information about cell lines

Cell line source(s)	HeLa (Kyoto, ATCC), HEK293-T (ATCC)
Authentication	Cell lines were not re-authenticated.
Mycoplasma contamination	Cell lines were routinely tested negative for mycoplasma contamination in-house.
Commonly misidentified lines<br>(See ICLAC register)	The cell lines were purchased from ATCC. No commonly misidentified cell lines were used.<br>The HeLa cell line is routinely used in our lab for the investigation of ADP-ribosylation.<br>HEK 293-T was used for the expression of the Fc fusion domains.
